# Stability and yield performance of lablab genotypes across multiple environments in Ethiopia

**DOI:** 10.1038/s41598-026-48048-7

**Published:** 2026-05-22

**Authors:** Melkam Aleme, Gezahegn Mengistu, Dereje Tulu, Mesfin Dejene, Fekede Feyissa, Shiferaw Temteme, Getnet Assefa

**Affiliations:** 1 Ethiopian Institute of Agricultural Research, Tepi Agricultural Research Center, P.O.Box:34, Tepi, Ethiopia; 2 Ethiopian Institute of Agricultural Research, Holeta Agricultural Research Center, P.O.Box:2003, Addis, Ababa Ethiopia; 3 Ethiopian Institute of Agricultural Research, P.O. Box, 2003, Addis Ababa, Ethiopia; 4 International Livestock Research Institute, P.O. Box, 5689, Addis Ababa, Ethiopia

**Keywords:** AMMI, ASV, Ethiopia, GGE, Yield, Genetics, Plant sciences

## Abstract

In Ethiopia, the availability of quality animal’s feed is a greater challenge to livestock production than the animal genetic resources and their health problems. In this context to identify suitable genotypes for particular environment, 13 Lablab genotypes (*Lablab purpureus* L.) were examined across three different sites of Ethiopia during the year of 2020 and 2021 for their performance on dry matter yield. A randomized complete block design (RCBD) with three replications in each location was applied to complete the trials. The dry matter yields were logged and analyzed using the additive main effects multiplicative interaction (AMMI) and genotype plus genotype -by-environment (GGE) biplot models. The combined analysis of variance showed that dry matter yield was significantly affected by the environment (63.04%), followed by genotype (15.90%), and genotype by environment (G×E) interface (10.04%). Based on the AMMI and GGE biplot, the tested genotypes were categorized into four mega-environments. The first comprises E3 and E6, the second comprises E4, the third comprises E1, and the last mega-environment holds E2 and E5. The GGE biplot analysis revealed that the six endearing genotypes, G1, G3, G7, G12, and G13, scored higher dry matter yield in corresponding environments, While the AMMI model showed the G5, G6, G9, G10, and G11 were comparably stable higher yielders, whereas G12 had the highest dry matter yield but was an unstable genotype. Based on the AEC line, genotypes namely G10, G6, G2, and G5 were relatively stable, though G13, G3, and G1 were unstable genotypes. According to this finding, breeding improvement was shown in dry matter, and the selected genotypes could be suggested for national production.

## Introduction

Lablab (*Lablab purpureus* L.) has a diploid chromosome number of 2n = 22 and is a historically common self-fertilized crop in the Leguminosae family of cultivated plants^1,2^. It has more potential as a crop than cowpea because of its better grain yields and more adaptability to different agro-ecological zones than other leguminous plants^3^. However, little effort has been put into species-wide drought adaptation^4^. Together with fibrous grass, Lablab helps balance and increases the nutritional needs of feed when it comes to providing nutrients to cattle. According to previous studies, ruminant animal performance is generally enhanced when fed fibrous crop leftovers and forage materials such maize Stover supplemented with Lablab fodder^5^. The foliage of crops yields hay, silage, and green manures, in addition to being utilized in various vegetable preparations^6^.

The additive main effects and multiplicative interaction (AMMI) technique combines principal component analysis and analysis of variance into a single strategy^7^. It can also be used to analyze multi-environment trials (METs)^8^, . There are three primary uses. Model diagnoses are the first, and AMMI is more appropriate for the initial statistical analysis of yield trials because it offers an analytical tool for diagnosing additional models as sub instances when they are enhanced for certain data sets^7^. Second, AMMI describes patterns and connections between genotypes and environments and clarifies the genotype environment interaction (GEI). The third often used and flexible in GEI studies was Principal component analysis (PCA)^8^.

When studying the relationship between genotypic and phenotypic characteristics, the G× E interaction limits the usefulness of genotypes by confusing their yield performance^9^. This complicates the process of varietal selection. However, by dividing a heterogeneous area into smaller, more homogenous sub-agroecology areas and choosing genotypes with higher stability across a wide variety of environments, it is possible to generate genotypes with minimal G×E interactions^10^. Therefore, breeders may view the G×E interaction as both chance and problem. Data from multi-environment trials should be analyzed using AMMI, which interprets the effects of the genotype (G) and the environment (E) as additive effects and G×E as a multiplicative component (which is sources of variation) before subjecting it to PCA. Since the AMMI approach fits additive main effects for genotypes and environments using a regular ANOVA procedure and then applies PCA to the matrix of residuals that are left after the fitting of main effects, it has been demonstrated to boost estimation accuracy^11^. The treatment plan of a yield trial serves two key goals of the AMMI analysis.

In multi-environment trials, the GGE biplot will help researcher’s better understand complex GE interactions^12^. The performance of crop cultivars under various stress conditions, ideal cultivars, mega-environment, and core testing sites; were assessed using the GGE biplot. Because this method only decomposes G×E interaction effects in PCA, it is not possible to directly depict genotype effects in the AMMI2 biplot. However, GGE biplot analysis is recognized as a practical statistical method for developing superior and phenotypically stable cultivars, discovering stable genotypes across various conditions, and attaining crop yield stability across many locations^13^.

In a preliminary observation screening trial run at Tepi Agricultural Research Center (TARC), 98 Lablab accessions, obtained from the International Livestock Research Institute (ILRI) forage diversity gene bank in Addis Abeba were assessed over the course of two years. The performance of the accessions’ forage and grain yields, as well as their resistance to disease in Tepi’s warm, muggy environment were assessed. Twelve accessions were selected from these initial screens for additional testing in a repeated trial to assess forage and grain production performance and disease tolerance. Plant breeders can use this diversity to increase the crop output. Before conducting evaluative biochemical and molecular research, morphological evolution and screening should first be completed^13^. Utilizing newly introduced forage legumes is a practical way to address the livestock feed shortage. Supplementing with forage legumes is a sustainable strategy to increase the nutritional value of low-quality crop waste and pastures, particularly for small farmers who lack access to resources. To solve this issue, more research must be conducted on how well modified fodder crops adapt to the local environment and how well they perform and stabilize in terms of forage biomass yield. Therefore, the current experiment was initiated to determine the effect of the G×E interaction and stability for dry matter yield of Lablab genotypes.

## Materials and methods

### Study area

The trials were carried out over two years (2020–2022) at three locations (Tepi, Bechi, and Kite, Table 1) and a total of six environments (Table 2) of South-West Ethiopia.

**Table 1:** Description of the study areas.

Explanation	Testing area		
	Tepi	Bechi	Kite
Latitude	7^0^19’ N	7^0^22’ N	6^0^95’ N
Longitude	35^0^42’ E	35^0^53’ E	35^0^51’ E
Altitude (m)	1200	1276	1200
Annual rain fall (mm)	1559	1574	1200
Annual temperature (^0^C)	16.09–30.23	16.5-35.25	15.1–27.5
Soil pH	6.3	5.9	5.1
Soil type	Clay	clay	Clay-loam

Laboratory work and map of experimental area on coordination system, 2023, Aleme et al.^15^.

**Table 2:** Codes used for investigational environments during 2020–2021 and 2021–2022 cropping season.

Year	Location		
	Tepi	Bechi	Kite
2020–2021	E1	E2	E3
2021–2022	E4	E5	E6

## Materials used

In this study, the 12 advanced genotypes and one check were evaluated for two consecutive years in three locations: Tepi, kite and Bechi in Sheka and Bench Sheko Zones of the Southern Peoples Nations and Nationalities (SNNP) region of Ethiopia (Table 3). The lablab variety used as a check was made available by the Bako Agricultural Research Center in 2017 and registered with the Ministry of Agriculture’s variety registration.

**Table 3:** Genotypes used for the experiment.

Code of genotypes	Characters	Accession number
G1	Accession	ILRI6528
G2	Accession	ILRI14425
G3	Accession	ILRI14435
G4	Accession	ILRI14459
G5	Accession	ILRI11619
G6	Accession	ILRI14445
G7	Accession	ILRI11614
G8	Accession	ILRI14417
G9	Accession	ILRI11612
G10	Accession	ILRI11615
G11	Accession	ILRI10953
G12	Accession	ILRI11613
G13	Check	Gebisa

### Field management

The study was conducted under rain-fed conditions without irrigation. After sowing, the experimental field was weeded manually for three consecutive months, and the pathways between blocks and plots were cleaned during each weeding.

## Estimation of dry matter yield

Dry matter yield was estimated by mowing the two inner rows of each plot of Lablab at 50% flowering stage^16^. Harvesting was performed manually using sickles at approximately 5 cm above ground level. After harvesting, samples were collected to determine dry matter percentage. A fresh sample of 300 g was weighed and oven-dried at 65 °C until a constant weight was obtained. The dry matter percentage was calculated by dividing the dry weight by the fresh weight and multiplying by 100^17^.

$$\:\mathrm{D}{M}{\%}=\:\frac{\mathrm{D}\mathrm{r}\mathrm{y}\:\mathrm{f}\mathrm{o}\mathrm{r}\mathrm{a}\mathrm{g}\mathrm{e}\:\mathrm{s}\mathrm{a}\mathrm{m}\mathrm{p}\mathrm{l}\mathrm{e}\:\mathrm{w}\mathrm{e}\mathrm{i}\mathrm{g}\mathrm{h}\mathrm{t}\:}{\mathrm{F}\mathrm{r}\mathrm{e}\mathrm{s}\mathrm{h}\:\mathrm{s}\mathrm{a}\mathrm{m}\mathrm{p}\mathrm{l}\mathrm{e}\:\mathrm{w}\mathrm{e}\mathrm{i}\mathrm{g}\mathrm{h}\mathrm{t}}\times\:100$$

The DM % was used to estimate dry matter yield per hectare.

DM forage yield (t/ha) = Total fresh yield in t/ha x DM%,

The fresh yield t/ha was calculated as,

$$\:\mathrm{F}\mathrm{r}\mathrm{e}\mathrm{s}\mathrm{h}\:\mathrm{f}\mathrm{o}\mathrm{r}\mathrm{a}\mathrm{g}\mathrm{e}\:\mathrm{y}\mathrm{i}\mathrm{e}\mathrm{l}\mathrm{d}\:(\mathrm{t}/\mathrm{h}\mathrm{a})=\:\frac{\mathrm{p}\mathrm{l}\mathrm{o}\mathrm{t}\:\mathrm{y}\mathrm{i}\mathrm{e}\mathrm{l}\mathrm{d}\:\mathrm{i}\mathrm{n}\:\mathrm{k}\mathrm{g}}{\mathrm{p}\mathrm{l}\mathrm{o}\mathrm{t}\:\mathrm{s}\mathrm{i}\mathrm{z}\mathrm{e}\:\mathrm{i}\mathrm{n}\:{m}^{2}}\times\:10$$

## Experimental design

A randomized complete block design (RCBD) in triplicate. 20 kg/ha of seeds were scattered at a rate of 14.4 g per plot^18^. Before planting, treatments were randomly assigned to each plot in a block. Plants were then planted on a 3 m × 2.4 m plot that had become 7.2 m^2^ with rows 40 cm apart and plants spaced 30 cm a part^19^. The blocks and plots were separated by 1.5 m and 1 m, respectively.

### Data collection

The dry matter yield was collected from the middle rows in one by one meter area and sampled in an oven dry at 65 °C for 72 h during 50% flowering period at 90 days after sowing, where Lablab is recommended for uses as a forage crop.

### Data analysis

R -software version 4.4.2 was used for data analysis. The data were examined using the additive main effects and multiplicative interaction (AMMI) model^20^. The AMMI model initially turns the additive effects for the primary effects of genotypes (G) and environment (E) before principal component analysis (PCA) fits the multiplicative effects for GEI. In the AMMI model, the interaction (GEI) and residual can be divided into several Interaction Principal Component Axes (IPCAs). The AMMI analysis’s results are shown as biplot graphs. The AMMI model with following formula^21^ was used for present analysis:

$$\:{y}_{ge}={\mu\:+a}_{g}+{\beta\:}_{e}+{\sum\:}_{n=1}^{N}\left({{\uplambda\:}}_{n}{y}_{gn}{\delta\:}_{en}+{\mathrm{Q}}_{ge},\right)$$

where $\:{y}_{ge}$ is the characteristic mean of genotype g in environment e; µ is the grand mean; α_g_ is the mean genotype deviation; β_e_ is the mean environmental deviation; N is the number of PCA axes engaged in the adjusted model; λ_n_ is eigenvalue of PCA axis n; γ_gn_ is the genotype score for PCA axis n; δ_en_ is score eigenvector for PCA axis n; and Q_ge_ is residual, including the AMMI noise and pooled experimental error. Genotype stability was assessed using the AMMI stability value (ASV) coefficient^22^;

$$ASV = \sqrt {{{[\frac{{S{S_{IPCA1}}y}}{{S{S_{IPCA2}}}}\left( {IPC{A_1}} \right)]}^2} + {{(IPC{A_2})}^2}}$$

Where, SS_IPCA1_ is the sum of squares for IPCA1, SS_IPCA2_ is the sum of squares for IPCA2, and IPCA1 and IPCA2 are the genotype scores in the AMMI model. The genotype was more stable under the examined conditions and the ASV value was lower. For each genotype, the genotype selection index (GSI), which was determined by adding the ASV and yield stability index (YSI) ranking positions^23^, was calculated for each genotype.

The GGE biplot allows visualization of any crossover G × E interaction and various visual meanings that additive main effects and multiplicative interaction do not. In most circumstances, the GGE biplot is comparable to the best additive main effects and multiplicative interaction models^24^. Additionally, the first principal component score, which indicates the genotypic level rather than the additive level, is more logically explained by the GGE biplot for biological purpose^25^. The first two main components were decomposed into singular values to form the basis of the model for a GGE biplot^26^. Yij – ì – aj = el îil çjl + e2 îi2 çj2 + εij; Where, Yij = measured mean of genotype i in environment j, Ì = grand mean, aj = key effect of environment j, ì+âj = mean yield across all genotypes in environment j, e1 and e2 = singular values for the first and second principal components, respectively, îi1 and îi2 = eigenvectors of genotype i for first and second principal components, respectively, ç1j and ç2j = eigenvectors of environment j for the first and second principal components, respectively, aij= residual associated with genotype i in environment j.

### Yield stability index

The mean yield and solidity were both included in the yield stability index as criteria. According to Bose et al.^27^. and Tumuhimbise et al.^28^., genotypes with low values of indices have desirable traits such as high mean yield and stability. The following formula was used to determine the yield stability index;

YSI= R^ASV^ + R^DMY^ Where, R^ASV^ = the ranking of the AMMI stability value, R^DMY^ = the ranking of lablab genotypes dry matter yields in all environments.

## Results

### Genotype by environment interaction

The additive main effect and multiplicative interaction (AMMI) showed that genotype and environment had (p *≤* 0.001) effect, and the interaction (G×E) significantly (p *≤* 0.05) affected the dry matter yield of Lablab genotypes (Table 4). Henceforth, the source of variation accounted for 15.90%, 63.04%, and 10.04% of the sum of squares for the genotypes, environments and genotype-environmental interaction (GEI) respectively.

The AMMI model showed that the mean square of the first principal component was highly significant (p *≤* 0.001) and the second principal component axis (p *≤* 0.05) but the third principal component axis (IPCA3) was not significant (*p* > 0.05). Likewise, IPCA1 and IPCA2 accounted 53.66 and 34.37% of the interaction sum of squares, respectively, capturing 87.03% of the total GEI. Furthermore, IPCA1 mean square was greater than the second and third IPCAs, which showed that dry matter yield was significantly affected by the GEI.

**Table 4:** AMMI ANOVA for dry matter yield (t ha^− 1^) of 13 lablab genotypes across six environments.

Source	DF	SS	MS	Sum of square explained (%)	G*E explained (%)	Cumulative explained (%)
Total	119	2380.94	25.33			
Genotypes (G)	12	378.60168	31.55***	15.90		
Environments (E)	5	1500.9698	300.19***	63.04		
G*E	60	239.06961	3.98*	10.04		
IPCA1	16	128.29456	8.02***		53.66	53.66
IPCA2	14	82.17687	5.87*		34.37	88.04
IPCA3	12	17.70695	1.48ns		7.41	
Error	139	0.0001	0.0001			
Residuals	156	450.3298	0.0001			

DF= degree of freedom, SS = sum squar, MS= mean squar, G8E= genotyp by environment.

### Mean of dry mater yield across six environments

The mean dry matter yield of Lablab varied significantly in each environment and was further ranked in terms of the environment. (Table 5; Fig. 2A). The average environmental dry matter yield ranged from 1.17 t ha^− 1^ at E3 to 14.20 t ha^− 1^ at E1 with grand mean of 7.66 t ha^− 1^. The performance of the genotype varied with variations in the environment.

**Fig. 1:**
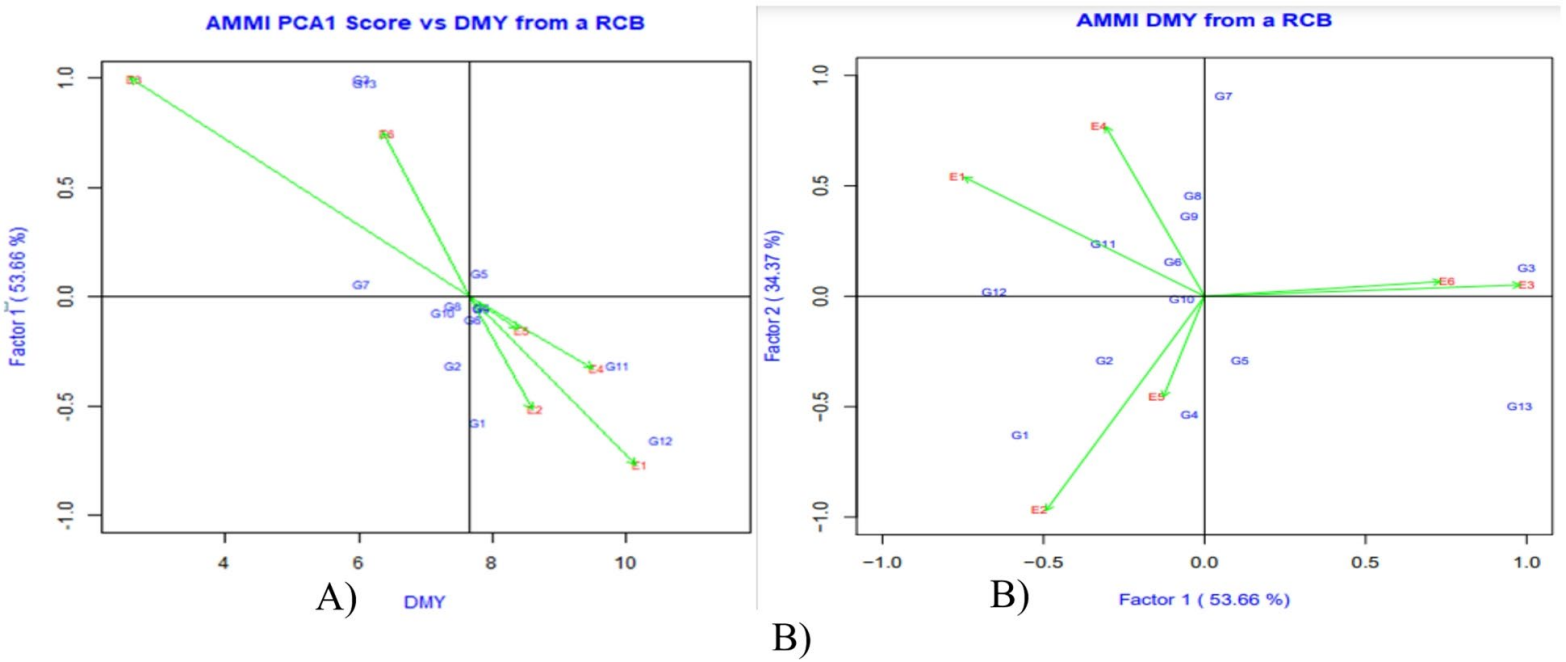
Thirteen lablab genotypes were used in the biplot “AMMI 1,” which showed the effects of (**a**) dry matter yield and the first principal component and (**b**) the first and second principal components.PCA= principal component analysis, DMY = dry matter yield, RCB= randomized complet block design.

Additive main effects and multiplicative interaction (AMMI1 and 2) indicated that the biplot abscissa and ordinate of 1st principal component (PC1) term and the dry matter yield showed significantly influenced. In the present study, based on the relationship between environment and genotype, little similarity was observed. Likewise, E2, E6, and E11 had little effect on the dry matter yield of Lablab genotypes (Fig. 1A). Based on AMMI2 using the principal component (PC1 and PC2) the short vector length E5 and E6 relatively other environments were less discriminating to select the genotype (Fig. 1B).

The E6 was the most discriminative and representative test environment, making it ideal for selecting adapted genotypes. Specifically adapted genotypes were selected using environment E2 and E4 which were discriminating and non-representative test environments however, E4 helped to cull unstable genotypes because it was a single mega-environment (Fig. 4B).

**Fig. 2:**
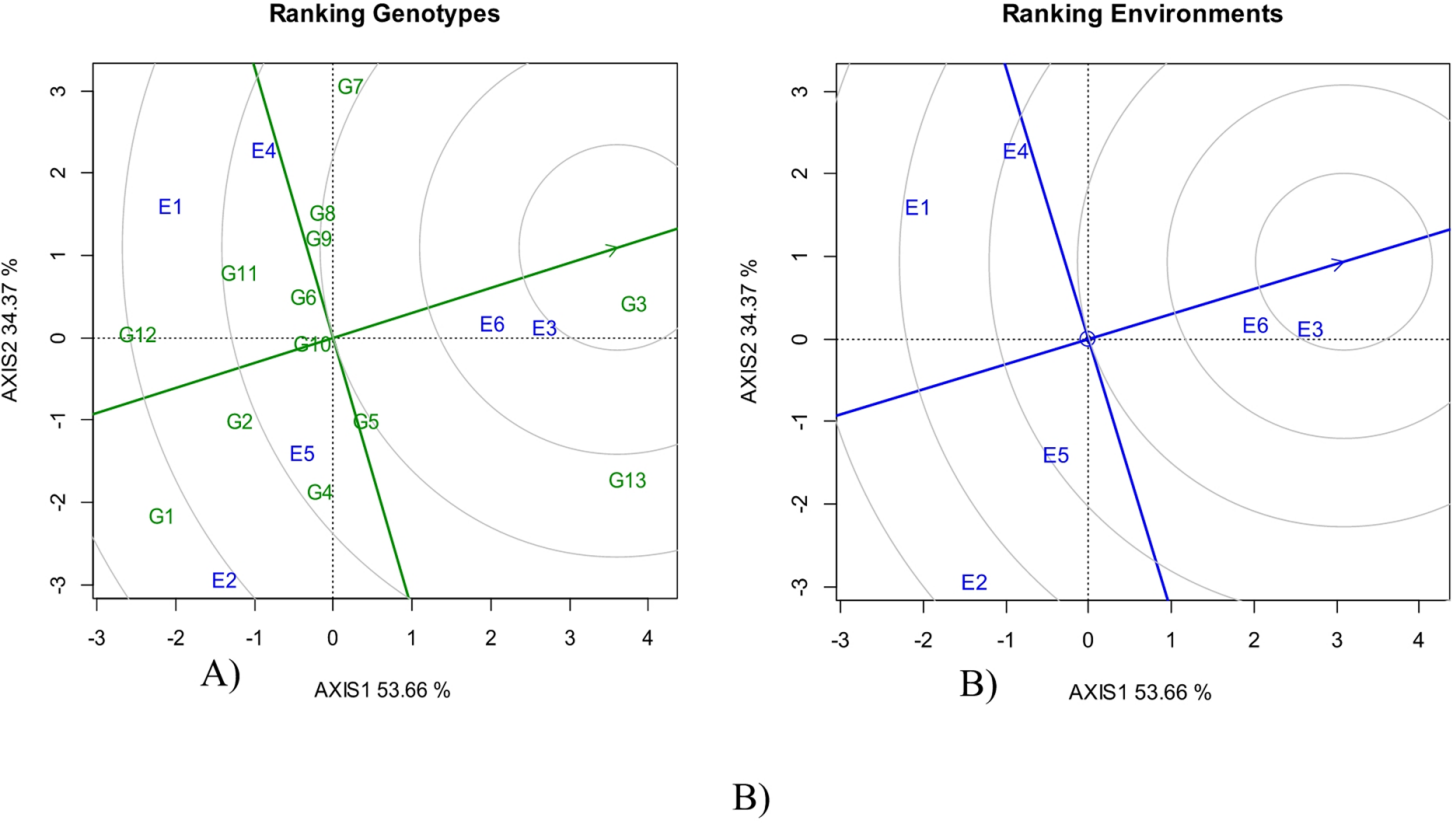
The effects of the G + GE interaction between 13 lablab genotypes in six environments for dry matter yield are shown by the GGE biplot **a**) ‘Environment ranking’ and **b**) “genotypes ranking” pattern for comparing the actual environment to the ideal environment.

**Fig. 3:**
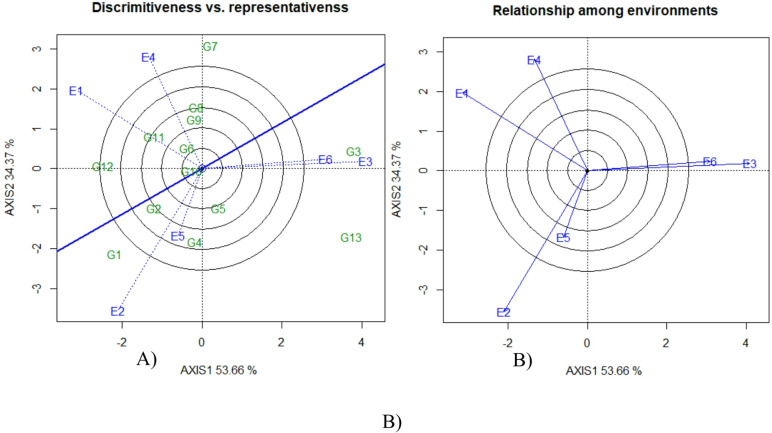
The GGE biplot **a**) Descriminitiveness vs. Representativeness and **b**) Relationship among environments pattern for genotype comparison with ideal environment shows G + GE interaction effect of thirteen lablab genotypes in six environments for dry matter yiel.

**Table 5:** Mean dry matter yield (t ha^− 1^) of 13 lablab genotypes in six environments and stability indicators of AMMI analysis.

Genotype	E1	E2	E3	E4	E5	E6	Mean	IPCAg1	IPCAg2	IPCAg3
G1	11.18^ab^	10.66^ab^	1.63^gf^	8.33^bcd^	9.67^abc^	5.20^e^	7.78^b^	-0.5711921	-0.6272211	-0.0851363
G2	9.87^bc^	9.51^abc^	1.71^gf^	9.33^bcd^	8.67^bcd^	5.33^e^	7.41^b^	-0.3106224	-0.2902298	-0.0339818
G3	7.07^cd^	5.70^de^	3.60^ab^	7.33^cd^	6.00^d^	6.57^cde^	6.03^d^	1.001	0.1284771	-0.178732
G4	9.55^bcd^	10.51^abc^	2.33^def^	9.07^bcd^	8.67^bcd^	6.87^bcd^	7.83^b^	-0.0460493	-0.5394714	-0.0211392
G5	9.63^bcd^	9.26^abcd^	2.67^cde^	9.17^bcd^	9.00^abc^	7.03^bc^	7.79^b^	0.1101801	-0.2904193	0.1983205
G6	10.77^ab^	8.71^bcd^	3.37^abe^	10.00^abc^	8.00^cd^	5.37^de^	7.81^b^	-0.0985589	0.1533216	-0.5273032
G7	9.83^bc^	4.70^e^	1.17^g^	9.33^bcd^	6.00^d^	5.20^e^	6.04^d^	0.0588021	0.9059573	0.1014862
G8	10.75^ab^	7.37^bcde^	2.27^def^	10.00^abc^	6.67^cd^	6.33^cde^	7.39^b^	-0.035867	0.4521412	0.0262339
G9	10.84^ab^	8.44^bcd^	3.03^bcd^	10.67^ab^	6.67^cd^	6.33^cde^	7.83^b^	-0.048116	0.3623008	-0.3056533
G10	10.06^bc^	8.18^bcde^	2.07^ef^	9.00^bcd^	8.33^bcd^	5.87^cde^	7.25^bc^	-0.070182	-0.0143904	0.0719072
G11	12.77^ab^	10.07^abc^	2.23^abc^	12.73^a^	11.67^a^	8.67^a^	9.86^a^	-0.3139179	0.237202	0.7558778
G12	14.20^a^	12.59^a^	3.97^a^	13.13^a^	11.00^ab^	8.17^ab^	10.51^a^	-0.653201	0.0203996	-0.1649517
G13	6.13^d^	6.81^cde^	3.27^abc^	6.33^d^	7.50^cd^	6.50^cde^	6.09^cd^	0.9787244	-0.4980677	0.1630719
Mean	10.2	8.65	2.64	9.57	8.45	6.42	7.66			
IPCAe1	-0.7664715	-0.5117605	1.00	-0.3266694	-0.1477185	0.7526198				
IPCAe2	0.5395198	-0.9698485	0.0512566	0.7683509	-0.4552904	0.0660117				
IPCAe3	-0.1879835	-0.3357396	-0.5293733	0.0517838	0.5811377	0.420175				

Means followed by the same column were non-significant at (*p* < 0.05).

### Stability and mega-environments

The additive main effect multiplicative interaction stability value (ASV), and yield stability index (YSI) ranked the genotypes based on least value of rank value (Table 6).

**Fig. 4:**
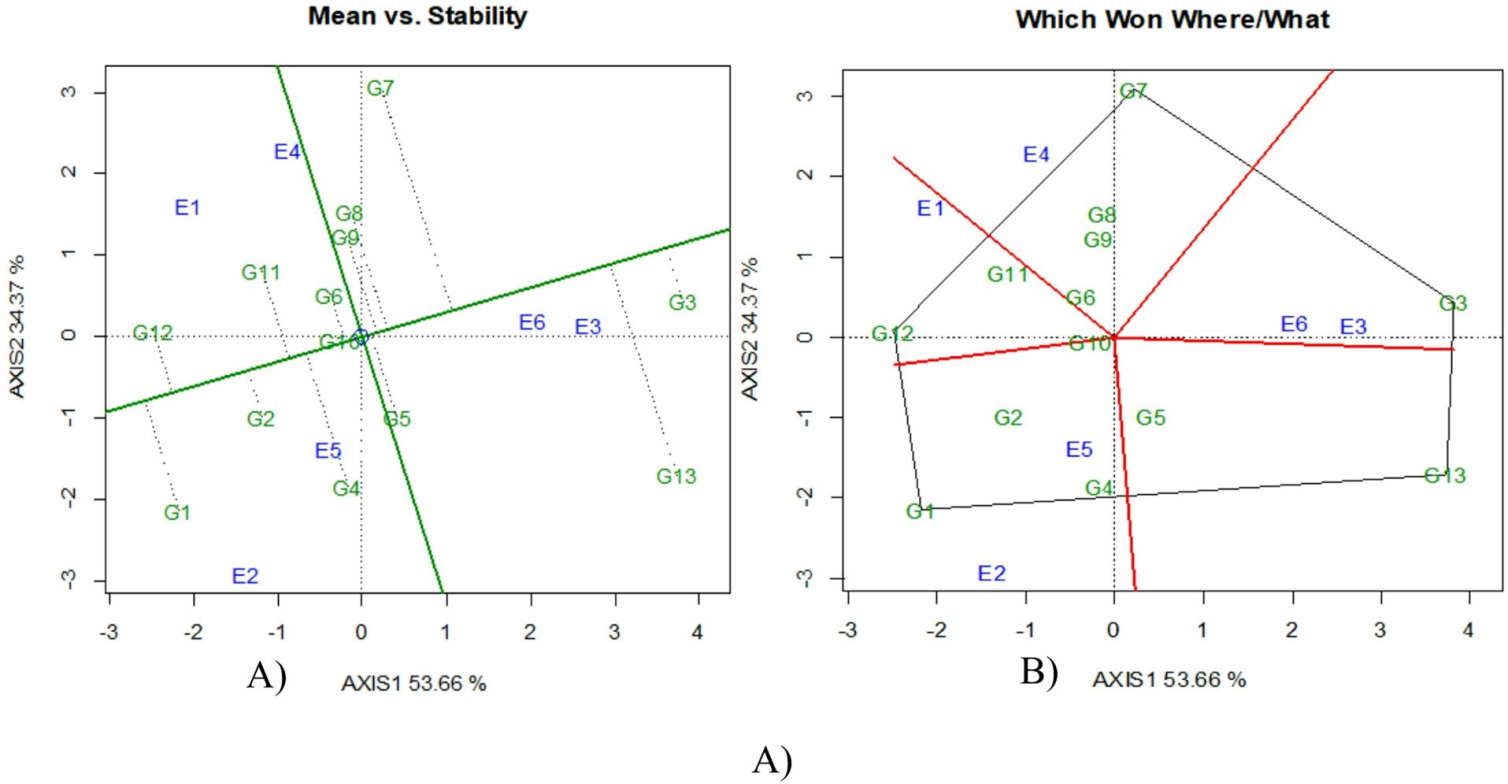
The GGE biplot (**a**) Mean vs. stability (**b**) Which won where/what pattern shows the interaction effect of thirteen lablab genotypes in six environments for dry matter yield.

The most interesting feature of the GGE biplot is the polygon view, which addresses the “which-won-where” pattern of multi-environment data and graphically illustrates the crossover GE interaction, mega-environment difference, and unique genotype adaptation. The genotypes that are farthest from the origin are connected to form a polygon that contains all additional genotypes (Fig. 4A). Therefore, vertex genotypes that were referred to as being on the edge were the most yielding genotypes in the given environment (Fig. 4B).

**Table 6:** AMMI stability value, ranking of dry matter yield IPCA1 and IPCA2 scores.

Genotype	IPCA1	IPCA2	ASV	*R* ^ASV^	DMY	*R* ^DMY^	YSI	*R* ^YSI^
G1	-0.57119	-0.62722	1.09	11	7.78	7	18	10
G2	-0.31062	-0.29023	0.32	3	7.41	8	11	5
G3	1.001	0.128477	1.56	12	6.03	13	25	13
G4	-0.04605	-0.53947	0.59	8	7.83	4	12	8
G5	0.11018	-0.29042	0.34	4	7.79	6	10	4
G6	-0.09856	0.153322	0.22	2	7.81	5	7	1
G7	0.058802	0.905957	0.91	9	6.04	12	21	11
G8	-0.03587	0.452141	0.46	6	7.39	9	15	9
G9	-0.04812	0.362301	0.37	5	7.83	3	8	2
G10	-0.07018	-0.01439	0.11	1	7.25	10	11	5
G11	-0.31392	0.237202	0.54	7	9.86	2	9	3
G12	-0.6532	0.0204	1.02	10	10.51	1	11	5
G13	0.978724	-0.49807	1.61	13	6.09	11	24	12
Grand mean					7.66			

ASV= AMMI stability value, R^ASV^= ranking of the AMMI stability value, DMY = Dry matter yield, R^DMY^ = Ranking of lablab genotypes dry matter yields in all environments, YSI= Yield stability index, R^YSI^= ranking yield stability index.

## Discussion

### Genotype by environment interaction

The present study demonstrated that dry matter yield of lablab genotypes was significantly influenced by genotype (G), environment (E), and genotype × environment interaction (GEI). The highly significant (*p* < 0.001) contribution of environments, accounting for 63.04% of the total variation, indicates that environmental factors were the primary drivers of yield variation (Table 4). This finding is consistent with previous reports in forage and grain legumes, where environmental heterogeneity such as rainfall distribution, soil fertility, and temperature plays a dominant role in determining productivity Guizani et al.^29^. who concluded that the environment was the highest source of variation. The relatively lower but significant contribution of genotypes (15.90%) suggests the presence of exploitable genetic variability among the tested lablab accessions.

The significant GEI (10.04%) further indicates that genotypes responded differently across environments, confirming the necessity of multi-environment trials for identifying stable and high-yielding genotypes. The significance of GEI implies that selection based on mean performance alone would be insufficient, and stability parameters must be considered. This result is in agreement with the earlier findings, Ashraf et al.^30^. for flax Tamene et al.^31^. for faba bean, and Ganta et al.^32^. for mung bean. In the present study the sum of squares of the first two IPCAs sum of accounted for more than 50% for the variations explained by GEI for the dry matter yield of Lablab genotypes which was validated in the AMMI model. This finding was in line with^33,34^ who showed a similar degree of GEI variance publicized by the first two principal components of GEI and indicated that AMMI with the first two multiplicative terms was the best predictive model. The non-significance of IPCA3 further supports the adequacy of using only the first two IPCAs for interpretation and graphical visualization.

### Mean of dry mater yield across six environments

The wide range of mean dry matter yield across environments (1.17 to 14.20 t ha⁻¹) highlights the strong environmental influence and variability in growing conditions. The superior performance of certain environments suggests favorable agro-ecological conditions, whereas low-yielding environments may have been constrained by limiting factors such as moisture stress or poor soil conditions. The variation in genotype performance across environments confirms the presence of crossover GEI, where genotypes perform inconsistently under different conditions. The mean dry matter yields of G12 and G11 were relatively higher across all environments and were 10.51 and 9.86 t ha^− 1^ respectively. The present study recorded a comparable dry matter yield of 7.2 t ha^− 1^ with Aleme^35^ in Lablab varieties. The significant variation in dry matter yield across environments suggests diverse weather conditions. Differences in soil type, temperature, and rainfall directly influenced genotype performance. However, the last two genotypes (G11 and G12) recorded relatively higher yields and the selection also considered the stability of the genotypes. The ideal and discriminating environments of the genotypes were found in E3 and E6 (Fig. 2B). At E3 and E6, the yields of G3 and G13 were higher, G7 at E4 and G1 at E2 and E5 (Fig. 2A) because the direction line indicated with the average tester axis (ATA) was in agreement with Ganta et al.^36^. for mung bean.

The distributions far apart from the origin of the genotypes showed variation in the environments in response to genotypes^37^. According to Oladosu et al.^38^., the angle between the genotype and the environment is the right angle, showing no correlation between the genotype and the environment. However, the larger and smaller angle between them showed negative and positive correlations, respectively.

The GGE biplot “which-won-where” pattern further revealed the presence of distinct mega-environments and specific adaptation of genotypes. Vertex genotypes identified in different sectors of the polygon were the best performers in their respective environments, confirming the presence of crossover GEI. This coordination showed that the shortest vector length near the origin or vertex of the biplot showed less discriminating environments, as hypothesized by Yan and Tinker^39^. Overall, the integration of AMMI and GGE biplot analyses proved effective in identifying both stable and high-yielding lablab genotypes as well as suitable test environments. The present study deduced that E3 and E6 were the least discriminative environments; however, E1, E2, E4, and E5 were the most discriminative or informative environments (Fig. 3A). The present study is in line with Ganta et al.^36^. in mung bean and Aleme et al.^17^. for Urochloa, who emphasized the importance of representative and discriminative environments in multi-environment trials. However, the smaller angle between the average environment axis (AEA) and the environment is more representative than the other test environments. Therefore, E5, and E6 were the most representative of the environments, whereas E2 and E4 were least representative.

Multi environment identification helps to identify the test environments that fix the environment discriminations and representativeness ability, inter-relationship, and representativeness of the tested sites. Hereafter, the angle between the two environments showed correlation among them^39^. In this study the acute angle between E3 and E6 as well as E2 and E5 showed a positive correlation with the angles formed as obtuse, and the right angle were showed negatively correlated for E4 with E5 and E2 with and E3 with E6 respectively; while uncorrelated environments were formed by E1 and E2 (Fig. 3B). The close associations between test environments provide the same information between genotypes, which helps to reduce the test environments and then possible to reduce operation cost, time and related logistics.

### Stability and mega-environments

The mean versus stability states that the short vector length near the average environment vertex is stable^39^. The genotype with the lowest ASV value was the most stable. Hence, the genotypes represented by G10, G6, G2, and G5 were relatively stable (Table 6; Fig. 4A). According to Farshadfar^23^, measurement of the most stable genotype is not always, or even relatively, the highest yielding genotype is not enough for selection in breeding programs considering both the stability and amount of yield. However, G13, G3, and G1 were unstable genotypes. On the other hand, the YSI deduced that smaller value raking genotypes were stable and had high yields. Therefore, genotypes G6, G9, G10, G11, and G5 were comparably stable and high yield genotypes. However, G13, G3, and G7 were unstable and had low yield genotypes. G12 had the highest dry matter yield, but was unstable because it had a high ASV value.

The equality line divides the biplot in to four sectors (mega-environments). However, two more sectors since Kocaturk et al.^40^ and Ramos et al.^41^ reported six sectors by dividing the biplot through equality lines in soya bean. However, according to Kindeya et al.^42^., two mega-environments are recognized for sesame. Hence, five genotypes were shown on the vertex as assigned; G12 found in E1, G7 for E4, G3, and G13 in E3, E6, and G1 to E2, and E5 had higher dry matter yields in the corresponding environments.

The present study demonstrates several methodological strengths, including the use of well-established multivariate analytical approaches (AMMI and GGE biplot) and the evaluation of genotypes across multiple environments, which enhances the understanding of genotype × environment interaction and yield stability. Additionally, the use of replicated field trials and standardized experimental procedures contributes to the reliability of the generated data. However, despite these strengths, some limitations remain that should be considered when interpreting the results.

First, the study focused solely on dry matter yield as the primary evaluation criterion. While yield is an important parameter, it does not fully capture the overall value of forage crops. Other important traits such as nutritional quality, digestibility, crude protein content, and resistance to biotic and abiotic stresses were not assessed, thereby limiting the comprehensiveness of the evaluation due to limited time and resources.

Finally, the study did not consider practical aspects such as economic feasibility, farmer preferences, and the suitability of genotypes under real farming conditions. The absence of such considerations limits the direct applicability and adoption of the findings at the farm level. Ieven if those limitation are listed the information presented in this finding is crucial for the generation of scientific procedures and results.

## Conclusion and recommendations

Dry matter yield of lablab genotypes in this study was strongly influenced by environmental factors, with genotype and genotype × environment interaction (GEI) also contributing significantly to the observed variation. The presence of GEI and crossover interactions indicates that genotype performance varied across the tested environments, highlighting the importance of multi-environment evaluation. Based on the AMMI and GGE biplot analyses, some genotypes showed relatively better stability and performance than others; however, these results should be interpreted with caution due to the limited number of test environments and genotypes. While genotypes such as G5, G6, G9, G10, and G11 exhibited comparatively stable performance, and G12 showed higher yield potential, further multi-location and multi-year evaluations are required before making definitive recommendations for breeding or large-scale production.

## Data Availability

The data is available from the corresponding author upon reasonable request.
